# Micro computed tomography images of capillary actions in rough and irregular granular materials

**DOI:** 10.1038/s41597-024-02925-w

**Published:** 2024-01-16

**Authors:** Sadegh Nadimi, Joao Mendes, Alejandro López, Laurenz Schröer, Sojwal Manoorkar, Sharon Ellman, Veerle Cnudde, Agostino Walter Bruno

**Affiliations:** 1 School of Engineering, Newcastle University, Newcastle upon Tyne, NE1 7RU UK; 2 Department of Mechanical and Construction Engineering, Northumbria University, Newcastle upon Tyne, NE1 8ST UK; 3 Faculty of Engineering, University of Deusto, Bilbao, Spain; 4 Department of Geology, Ghent University, Krijgslaan 281/S8, 9000 Ghent, Belgium; 5 Department of Earth Sciences, Utrecht University, Princetonlaan 8a, 3584 CB Utrecht, Netherlands; 6 Department of Civil, Chemical and Environmental Engineering, University of Genoa, 16145 Genoa, Italy

**Keywords:** Civil engineering, Hydrogeology, Applied physics

## Abstract

The present work investigates the effect of both surface roughness and particle morphology on the retention behaviour of granular materials via X-ray micro-computed tomography (µCT) observations. X-ray µCT images were taken on two types of spherical glass beads (i.e. smooth and rough) and two different sands (i.e. natural and roughened). Each sample was subjected to drainage and soaking paths consisting in a multiphase ‘static’ flow of potassium iodine (KI) brine (wetting phase) and dry air (non-wetting phase). Tomograms were obtained at different saturation states ranging from fully brine saturated to air dry conditions with 6.2 μm voxel size resolution. The data acquisition and pre-processing are here described while all data, a total of 48 tomograms, are made publicly available. The combined dataset offers new opportunities to study the influence of surface roughness and particle morphology on capillary actions as well as supporting validation of pore-scale models of multiphase flow in granular materials.

## Background & Summary

Capillary actions are known to strongly influence the strength and flow properties of granular materials^1–4^, thus playing a fundamental role in several natural and engineering applications (e.g. slope stability^5^, earth construction^6^, building foundations^7^, caking in pharmaceutics^8^ and agriculture^9^ to name a few). These actions rise at the interfacial equilibrium (i.e. meniscus) between vapour and liquid phases within the pore network of granular materials and they are influenced by both the surface roughness and morphology of the grains.

However, most of the existing models on capillary phenomena in granular and porous materials rely on the assumptions of spherical and smooth particles^10,11^. As the capillary forces acting on spherical particles at the liquid-vapour interface can be described by the Young-Laplace equation, numerous numerical solutions have been developed based on the above simplifying assumptions^12–14^. Some studies have modelled capillary actions in multiphase sharp-edged particles^15,16^, but the boundary conditions for the contact angle at the sharp edges require additional assumptions that hinder the direct application of the Young-Laplace equation.

Only a handful of models have attempted to reproduce the effect of particle shapes and surface roughness on capillary actions rising within the porous network of granular materials^17^. However, a lack of experimental data on the retention behaviour of rough and irregular granular materials is hindering the further development of accurate analytical and numerical capillarity models^18^.

**Fig. 1:**
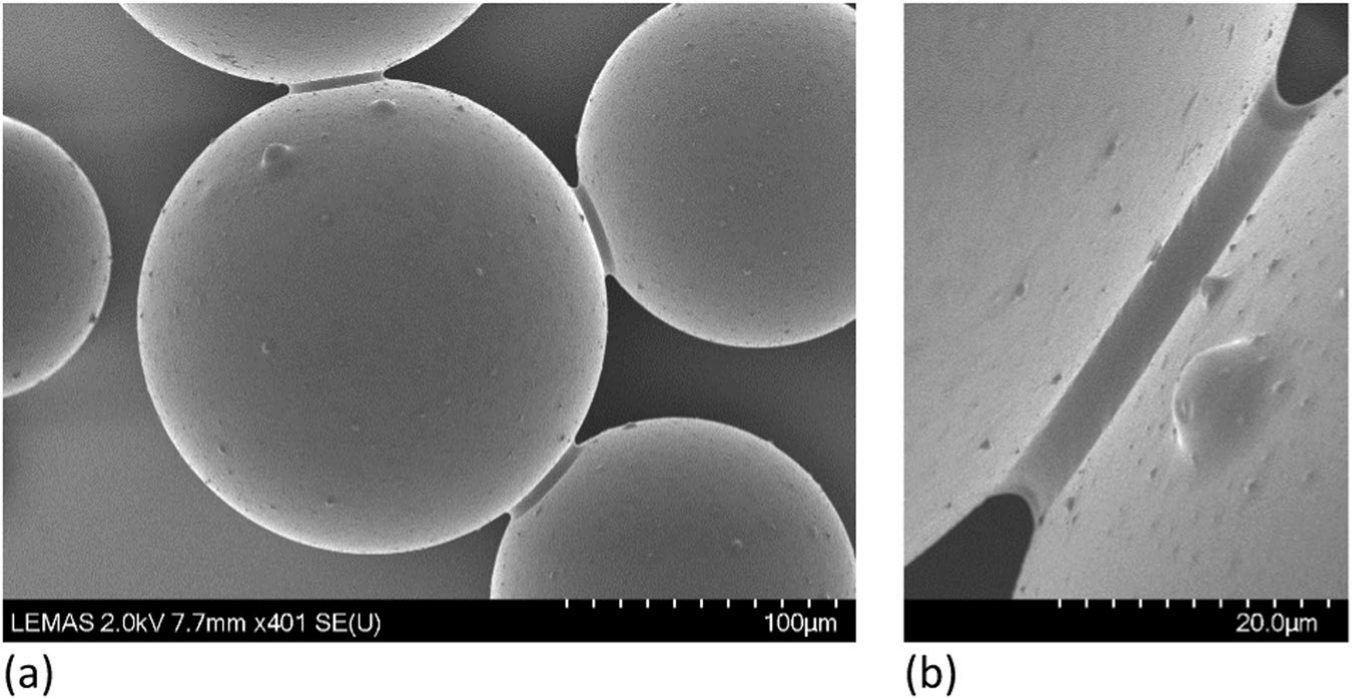
Scanning electron microscopy showing water menisci at inter-particle contacts of glass beads with a scale bar unit of 100 μm (**a**) and 20 μm (**b**).

Interestingly, recent advances in imaging techniques allow for an unprecedented visualisation of liquid menisci forming in multiphase granular materials. For instance, Fig. 1 shows a Scanning Electron Microscopy (SEM) image of water menisci forming at inter-particle contacts between glass beads (scanned at LEMAS centre at the University of Leeds). By taking advantage of these advanced imaging tools, the present work provides the first experimental dataset investigating the effect of particle morphology and surface roughness on the onset of capillary menisci in granular materials. For this purpose, four types of granular materials (namely smooth and rough spherical glass beads, natural and roughened sands) have been subjected to drainage (drying) and soaking (wetting) paths during which X-ray micro-computed tomograms were taken at different saturation states, ranging from fully brine saturated to air dry conditions. The resulting datasets are made available online^19–22^ with the aim of further animating ongoing research from the scientific community on the influence of surface roughness and particle morphology on a broad range of capillary state variables, such as water, air and solid volumes (i.e. phases distribution), contact lines, contact angles, radii of curvature, interfacial areas (i.e. air-brine and brine-solid interfaces) as well as other features, such as sphericity of air bubbles, pore-scale processes and grain movements.

## Methods

### Materials and sample preparation

X-ray micro computed tomography (µCT) scans were performed on smooth and rough spherical glass beads as well as natural and roughened sands to investigate the effect of surface roughness and particle morphology on the retention behaviour of granular materials. Both sands have a specific gravity of solid particles of 2700 kg/m^3^ while glass beads exhibited a lower value of 2500 kg/m^3^. All types of granular materials tested in the present work have a median particle size (d_50_) of 500 μm and they are mainly composed by silica. The surface of both sand particles and glass beads were mechanically roughened by milling the supplied materials following the technique described in the literature^23–25^. Measured by optical interferometry, the generated roughness root mean square height is in the range of 250–500 nm (<0.1% d_50_). The sphericity is 0.95 for the glass bead and is 0.86 for the sand. A dry mass of about 1.25 g for each tested material was measured by means of a balance with a resolution of 10^−4^ g and then inserted inside a testing tube. Inside this tube, each sample is sandwiched between a saturated high air entry value (HAEV) ceramic disc at the bottom and a metallic mesh at the top. The latter was needed to prevent sample particles from entering the drainage circuits and causing blockages. All samples had a diameter of 6 mm (i.e. nominal inner diameter of the testing tube) while the height was fixed at around 30 mm to target a value of dry density of about 1470 kg/m^3^. Sample height was measured by means of a calliper with a resolution of 10^−2^ mm. Table 1 summarises sample mass and dimensions as measured from both macroscopic measurements and µCT scans. The latter assume the same diameter of the macroscopic measurements and the height as the product of a voxel size of 6.2 μm and 1200 slices. Note that the sample height from the two measurements is different because µCT scans are only taken on a small mid-height portion of each sample. Table 2 lists instead the main physical properties (i.e. bulk and dry densities, void ratio and porosity) of each tested sample as determined from the macroscopic dimensions of Table 1. After insertion inside the testing tube, samples were subjected to drainage and soaking tests, as detailed in the following section.

**Table 1:** Samples mass and dimensions: macroscopic and microscopic determinations.

Sample	Dry mass [g]	Macroscopic measurements			Microscopic measurements (from µCT scans)		
		Height [mm]	Diameter [mm]	Volume [mm^3^]	Height [mm]	Diameter [mm]	Volume [mm^3^]
**Natural sand**	1.255	28.10	6.0	795	7.44	6.0	210
**Roughened sand**	1.254	30.17		853			
**Smooth spherical glass beads**	1.245	30.12		852			
**Rough spherical glass beads**	1.246	29.96		847

**Table 2:** Physical properties of all samples.

Sample	Bulk volume [mm^3^]	Dry density [kg/m^3^]	Void ratio [-]	Porosity [-]
**Natural sand**	795	1579	0.710	0.415
**Roughened sand**	853	1470	0.836	0.455
**Smooth spherical glass beads**	852	1462	0.710	0.415
**Rough spherical glass beads**	847	1471	0.699	0.411

### Drainage and soaking tests

**Fig. 2:**
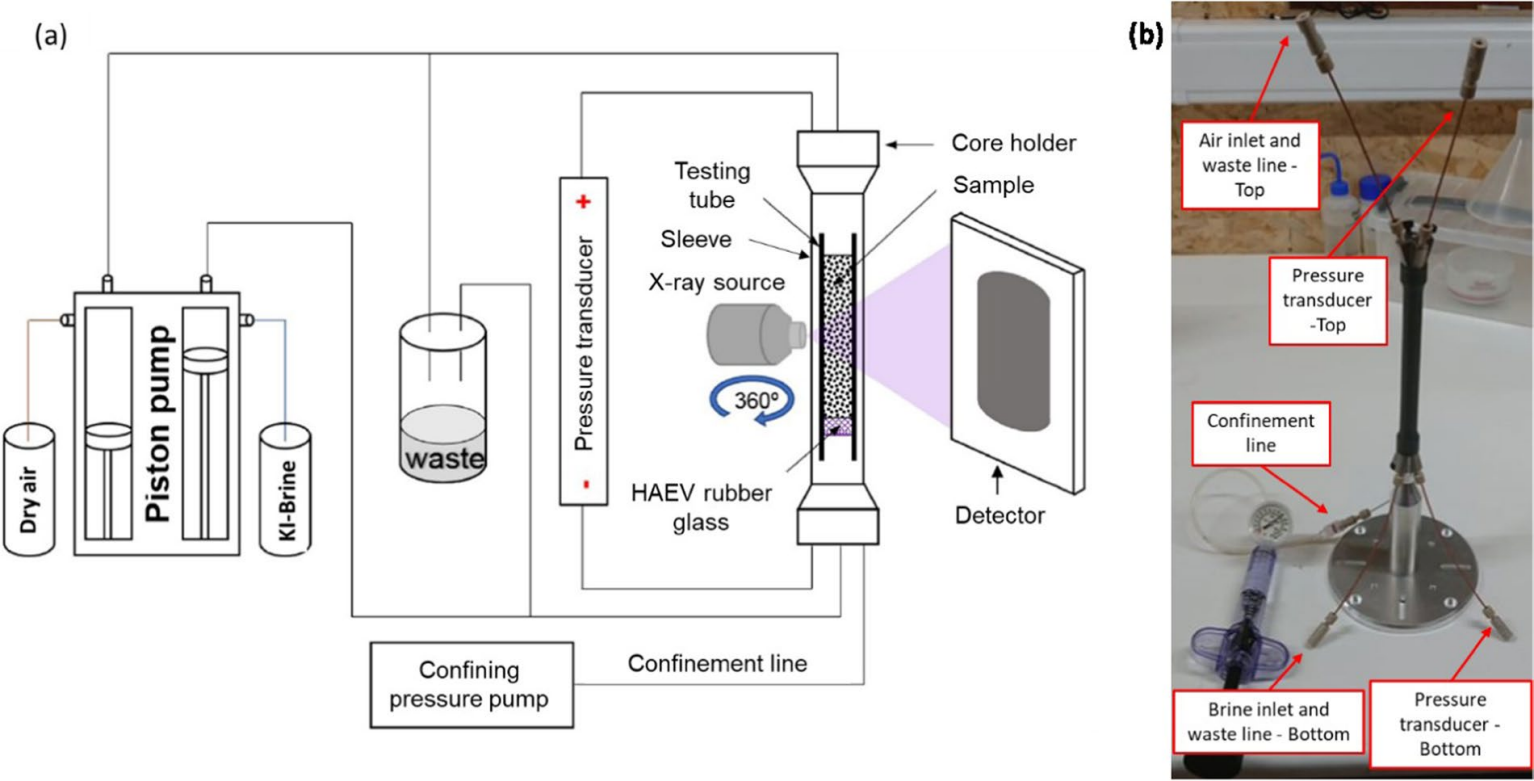
Schematic of the *in situ* µCT flow test set-up^26^ (**a**) and assembled roughened sand sample together with flow lines connections (**b**).

After sample preparation, the testing tube containing the sample was inserted inside a vitton sleeve that enabled the application of a confining pressure of about 1.5 MPa by means of a Reaxus pump. The sleeve was then inserted into a core holder and the two extremities of the sample were connected to (i) a dual-piston pump from Vindum Engineering for brine or air injection/extraction and waste lines for drainage, (ii) a pressure transducer measuring differential pressure between air and brine and (iii) a confinement pressure line. Figure 2 shows both a schematic of the testing setup, which is similar to that adopted by Wang *et al*.^26^ (Fig. 2a) and the sample assembly together with brine inlet, air inlet, waste-lines (bottom and top) and confinement line (Fig. 2b). After assembly, the sample was lifted into position by means of a vertical motor and centred between the X-ray source and the detector before starting the µCT tests. Initially, two tomograms were taken on each sample under air dry and deionised water saturated conditions. These first two scans will serve for the segmentation and subsequent 3D reconstruction of the solid particles (i.e. sand grains and glass beads). Afterwards, each sample was saturated with brine prepared by mixing deionised water either at 7.5% or 5% concentration by mass of potassium iodine (KI), as shown in Table 3. These concentrations were selected because they gave an optimal contrast of the three different phases (grains, air and brine). Note that increasing the brine concentration from 5% to 7.5% changed the solution density from 1036 to 1054 kg/m^3^, the surface tension from 73.15 to 73.32 mN/m and the relative viscosity respect to distilled water at 20 °C from 0.984 to 0.978, as determined from experimental data published in the literature^27–29^. Afterwards, a scan was taken on the brine saturated sample before dry air (non-wetting fluid) or brine (wetting fluid) were either injected or extracted at a constant flow rate of 0.005 mL/minute to perform the desiccation and soaking paths. Tomograms were then regularly taken as capillary menisci started to form until samples were either almost completely air dried or brine saturated with only a few isolated menisci. Tables 3 and 4 illustrates the consecutive sequence of desiccation and soaking paths applied to both sands and glass beads samples, respectively. Only in one case, due to testing time extending over a two-day period, testing conditions were repeated as in the previous tomogram (i.e. for tomograms named ‘10.NaturalSand_Test10’ and ‘11.NaturalSand_Test11’). Between these two tomograms no further injections or extractions of dry air or KI brine were imposed, and these tomograms serve only the purpose of verification of equalisation of the differential pressure between air and brine after the overnight test pause. For all other cases, as the testing was completed on a single day, each tomogram refers to a successive testing step compared with the precedent one. Moreover, all types of flow occurred vertically with air injected from the top of the sample in the downward direction while brine was either injected upwardly or extracted downwardly from the bottom of the sample, as it can be deduced from Fig. 2. Note that volumes of injected or extracted fluid indicated in Tables 3 and 4 do not correspond to the fluid volumes entering or exiting the sample portion analysed via µCT scans. This is because it is not possible to have accurate measurements of the injected air volumes because of the compressibility of this fluid. Moreover, a fraction of the fluid volumes is needed to soak or drain the sample extremities which are not included in µCT scans. Hence, an accurate estimate of the brine and air volumes within the scanned part of the samples can only be obtained from the tomograms^19–22^. Tables 3 and 4 also include measured values of differential pressure between air and brine at the two extremities of the sample.

**Table 3:** Drainage and soaking paths applied to both natural and roughened sands.

	File name^19,20^	Testing condition	Diff. pressure (Pa)
Natural sand	0.NaturalSand_Skeleton	Air dry sample	—
	1.NaturalSand_Dry	Sample saturated with deionised water	—
	2.NaturalSand_Wet	Sample saturated with brine at 5% concentration	—
	3.NaturalSand_Test1	Drainage by imposing a differential pressure of 8 kPa. Sample dried out completely	8000
	4.NaturalSand_Test2	Soaking by injecting 0.2 mL of brine	600
	5.NaturalSand_Test3	Soaking by injecting 0.05 mL of brine	400
	6.NaturalSand_Test4	Soaking by injecting 0.05 mL of brine	200
	7.NaturalSand_ Wet2	Sample saturated with brine at 5% concentration	0
	8.NaturalSand_Test8	Drainage by injecting 0.25 mL of air	450
	9.NaturalSand_Test9	Drainage by injecting 0.05 mL of air	600
	10.NaturalSand_Test10	Drainage by injecting 0.05 mL of air	1000
	11.NaturalSand_Test11	Same condition as ‘10. NaturalSand_Test10’	1000
	12.NaturalSand_Test12	Drainage by injecting 0.025 mL of air	1900
	13.NaturalSand_Test13	Drainage by injecting 0.025 mL of air	2400
	14.NaturalSand_Test14	Drainage by injecting 0.025 mL of air	2300
Rough sand	2.0.RoughSand_Skeleton	Air dry sample	—
	2.1.RoughSand_Dry	Sample saturated with deionised water	—
	2.2.RoughSand_Wet	Sample saturated with brine at 7.5% concentration	—
	2.3.RoughSand_Test3	Drainage by injecting 0.25 mL of air	0
	2.4.RoughSand_Test4	Drainage by injecting 0.05 mL of air	0
	2.5.RoughSand_Test5	Drainage by injecting 0.025 mL of air	1100
	2.6.RoughSand_Test6	Drainage by injecting 0.025 mL of air	1260
	2.7.RoughSand_Test7	Drainage by injecting 0.025 mL of air	1400
	2.8.RoughSand_Test8	Drainage by injecting 0.025 mL of air	1350
	2.9.RoughSand_Test9	Drainage by injecting 0.025 mL of air	1300
	2.10.RoughSand_Test10	Drainage by injecting 0.025 mL of air	1300

**Table 4:** Drainage and soaking paths applied to both smooth and rough spherical glass beads.

	File name^21,22^	Testing condition	Diff. pressure (Pa)
Smooth spher. glass beads	3.0.SmoothGlassBeads_Skeleton	Air dry sample	—
	3.1.SmoothGlassBeads_Dry	Sample saturated with deionised water	—
	3.2.SmoothGlassBeads_Wet	Sample saturated with brine at 7.5% concentration	—
	3.3.SmoothGlassBeads_Test8	Drainage by injecting 0.5 mL of air. No changes observed and test switched to brine extraction	—
	3.4.SmoothGlassBeads_Test9	Drainage by extracting 0.125 mL of brine	700
	3.5.SmoothGlassBeads_Test10	Drainage by extracting 0.05 mL of brine	976
	3.6.SmoothGlassBeads_Test11	Drainage by extracting 0.025 mL of brine	1020
	3.7.SmoothGlassBeads_ Test12	Drainage by extracting 0.025 mL of brine	1020
Rough spher. glass beads	4.0.RoughGlassBeads_Skeleton	Air dry sample	—
	4.1.RoughGlassBeads_Dry	Sample saturated with deionised water	—
	4.2.RoughGlassBeads_Wet	Sample saturated with brine at 7.5% concentration	—
	4.3.RoughGlassBeads_Test1	Drainage by injecting 0.2 mL of air. Sample dried out completely	1100
	4.4.RoughGlassBeads_Wet2	Sample re-saturated with brine at 7.5% concentration	700
	4.5.RoughGlassBeads_Test2	Drainage by injecting 0.1 mL of air	1100
	4.6.RoughGlassBeads_Test3	Drainage by injecting 0.025 mL of air	1200
	4.7.RoughGlassBeads_Test4	Drainage by injecting 0.075 mL of air	2300
	4.8.RoughGlassBeads_Test5	Drainage by injecting 0.025 mL of air	2200
	4.9.RoughGlassBeads_Test6	Soaking by injecting 0.025 mL of brine	1900
	4.10.RoughGlassBeads_Test7	Soaking by injecting 0.0125 mL of brine	1670
	411.RoughGlassBeads_Test8	Soaking by injecting 0.0125 mL of brine	1430
	4.12.RoughGlassBeads_Test9	Soaking by injecting 0.0125 mL of brine	1360
	4.13.RoughGlassBeads_Test10	Soaking by injecting 0.025 mL of brine	1230

### Imaging Setup

A laboratory-based µCT system was used for all experiments. µCT is a non-destructive technique to image internal structures and dynamic processes in opaque materials. The emitted X-rays are scattered and absorbed by the sample, which causes X-ray attenuation. This is controlled by the X-ray energy and the absorbing material’s density and atomic number^30^. In the present work, all µCT scans were taken using the EMCT system located at the Centre for X-ray Tomography (UGCT) at Ghent University. This gantry-based system is designed for *in situ* imaging, where the sample remains stable and the X-ray source and detector rotate around the sample, as in Dierick *et al*.^31^. The scans were taken at 90 kV and 8 W, with an exposure of 120 ms and no filter was applied. Each projection consisted of four averages while 2001 projections were made per scan, which lasted for about 16 minutes.

### Image reconstruction and processing

**Fig. 3:**
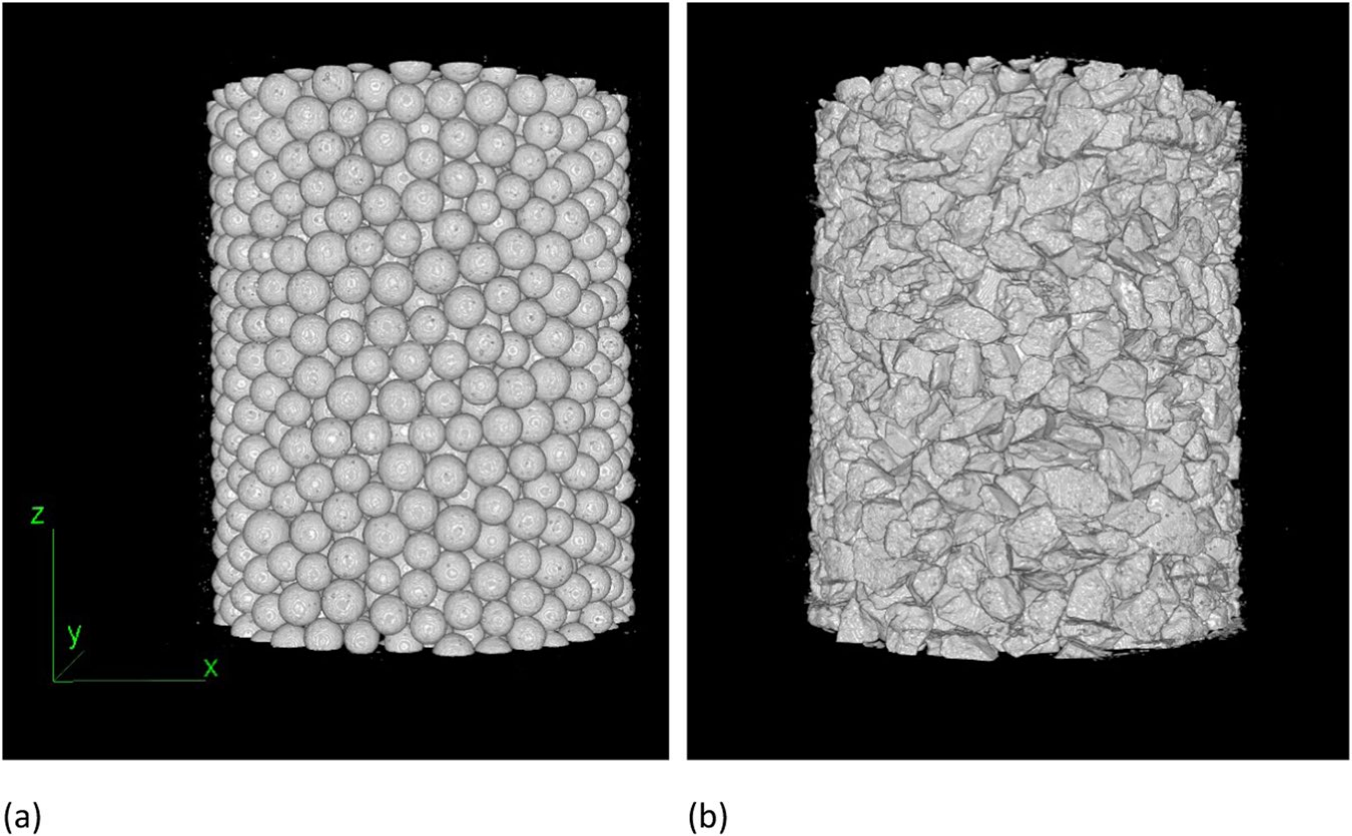
3D skeleton of glass beads and rough sand samples both with a median particle size of 500 μm.

The scans were reconstructed using Octopus Reconstruction version 8.9.4.9 (XRE). Beam hardening and ring filtering were applied. The reconstruction parameters and final grey values were kept constant for each sample to allow comparison of the scans. An extra ring filter was applied in some instances, if necessary. The reconstruction resulted in 16-bit cross-sections (.tiff files) through the sample with a final voxel size of 6.2 µm. 3D images of the skeleton of glass beads and rough sand are illustrated in Fig. 3. These images are obtained after denoising the raw images by first removing the dark spots with a size equal or smaller than 3 pixels and then the bright ones with the same size. For the purpose of technical validation, the samples are then binarised by using Otsu thresholding to estimate the preliminary 3D threshold value. This also served to calculate void ratio and porosity, as shown in the Section ‘Technical Validation’.

## Data Records

All the data records are publicly available on Zenodo repository. The dataset is comprised of 15 scans for natural sand, 11 scans for rough sand, 8 scans for smooth glass beads and 14 scans for rough glass beads. Testing conditions were instead summarised in Tables 3 and 4 for natural and rough sands and smooth and rough glass beads, respectively. The DOIs are listed below:

Natural Sand^19^: 10.5281/zenodo.8073680

Roughened Sand^20^: 10.5281/zenodo.8074919

Smooth glass beads^21^: 10.5281/zenodo.8075767

Rough glass beads^22^: 10.5281/zenodo.8073825

## Technical Validation

For the reconstructed images, the voxel size is 6.2 µm. However, the true image resolution refers to the finest detail distinguishable in the image, which depend on the voxel size, image blur and other artefacts. There is no universal measure that fully characterizes the true resolution. However, the Fourier ring correlation has been proposed as a fully automatic quantitative image-based measure without the need for prior information. This parameter is calculated using the Fourier Ring Correlation plugin in Fiji (ImageJ) on 2D slices of 3 different µCT images from the experiments^32,33^. This resulted in an average image resolution of 15.43 µm with a standard deviation of 0.49 µm. Table 5 compares the void ratio and porosity from both the macroscopic and the µCT measurements. Note that µCT scans are taken on a small mid-height portion of each sample under a confining pressure of 1.5 MPa. Hence, the discrepancies between the two measurements may have been generated by both edge effects and confinement. Despite these experimental constraints, the two macro- and micro-measurements of void ratio and porosity are indeed very similar, which confirms the good quality of the all the four datasets.

**Table 5:** Comparison of void ratio and porosity from macroscopic and microscopic measurements.

Sample	Void ratio		Porosity	
	Macroscopic measurement	Microscopic measurement (from µCT scans)	Macroscopic measurement	Microscopic measurement (from µCT scans)
Natural sand	0.710	0.703	0.415	0.413
Roughened sand	0.836	0.806	0.455	0.446
Smooth spherical glass beads	0.710	0.716	0.415	0.417
Rough spherical glass beads	0.699	0.742	0.411	0.426

## Data Availability

Image analysis is performed using the open-source software Fiji-ImageJ (1.53c) and MATLAB (R2021a). Shape characterisation of particles and calculation of shape descriptor parameters including surface roughness and sphericity were performed utilising the SHAPE code by Angelidakis *et al*.^34^ publicly available from the link below: https://github.com/vsangelidakis/SHAPE.

## References

[1] Scholtes L, Chareyre B, Nicot F, Darve F (2009). Micromechanics of granular materials with capillary effects. International Journal of Engineering Science.

[2] Gröger T, Tüzün U, Heyes DM (2003). Modelling and measuring of cohesion in wet granular materials. Powder Technology.

[3] Richefeu V, El Youssoufi MS, Radjai F (2006). Shear strength properties of wet granular materials. Physical Review E.

[4] Richefeu V, El Youssoufi MS, Peyroux R, Radjai F (2008). A model of capillary cohesion for numerical simulations of 3D polydisperse granular media. International Journal for Numerical and Analytical Methods in Geomechanics.

[5] Liang H, He S, Jiang Y (2021). Study of the dilatancy/contraction mechanism of landslide fluidization behavior using an initially saturated granular column collapse simulation. Water Resources Research.

[6] Pickert G, Weitbrecht V, Bieberstein A (2011). Breaching of overtopped river embankments controlled by apparent cohesion. Journal of Hydraulic Research.

[7] Peng Y, Yin ZY, Zhou C, Ding X (2023). Micromechanical analysis of capillary suction effect on bearing capacity of unsaturated fine granular foundation soil using coupled CFD-DEM method. Computers and Geotechnics.

[8] Zafar U, Vivacqua V, Calvert G, Ghadiri M, Cleaver JS (2017). A review of bulk powder caking. Powder Technology.

[9] Balzano B (2021). REAL-TIME quality check of measurements of soil water status in the vadose zone. Physics and Chemistry of the Earth, Parts A/B/C.

[10] Fisher RA (1926). On the capillary forces in an ideal soil; correction of formulae given by WB Haines. The Journal of Agricultural Science.

[11] Sun X, Sakai M (2016). Direct numerical simulation of gas-solid-liquid flows with capillary effects: An application to liquid bridge forces between spherical particles. Physical Review E.

[12] Princen HM (1969). The equilibrium shape of interfaces, drops, and bubbles. Rigid and deformable particles at interfaces. Surface and Colloid Science.

[13] Zhang L, Ren L, Hartland S (1996). More convenient and suitable methods for sphere tensiometry. Journal of Colloid and Interface Science.

[14] Zhang L, Ren L, Hartland S (1997). Detailed analysis of determination of contact angle using sphere tensiometry. Journal of Colloid and Interface Science.

[15] Hesla TI, Joseph DD (2004). The maximum contact angle at the rim of a heavy floating disk. Journal of Colloid and Interface Science.

[16] Singh P, Joseph DD (2005). Fluid dynamics of floating particles. Journal of Fluid Mechanics.

[17] Butt HJ, Kappl M (2009). Normal capillary forces. Advances in colloid and interface science.

[18] Shang J, Flury M, Deng Y (2009). Force measurements between particles and the air‐water interface: Implications for particle mobilization in unsaturated porous media. Water Resources Research.

[19] Bruno AW, Mendes J, Lopez A, Nadimi S (2023). Zenodo.

[20] Nadimi S, Mendes J, Lopez A, Bruno AW (2023). Zenodo.

[21] Mendes J, Nadimi S, Bruno AW, Lopez A (2023). Zenodo.

[22] Lopez A, Bruno AW, Nadimi A, Mendes J (2023). Zenodo.

[23] Cavarretta I (2012). Characterization of artificial spherical particles for DEM validation studies. Particuology.

[24] Otsubo, M.: Particle scale analysis of soil stiffness and elastic wave propagation. Ph.D. Thesis. Imperial College London (2016).

[25] Nadimi S, Otsubo M, Fonseca J, O’Sullivan C (2019). Numerical modelling of rough particle contacts subject to normal and tangential loading. Granular Matter.

[26] Wang S, Ruspini LC, Øren PE, Van Offenwert S, Bultreys T (2022). Anchoring multi‐scale models to micron‐scale imaging of multiphase flow in rocks. Water Resources Research.

[27] Apelblat A, Manzurola E (2005). Volumetric and thermal properties of some aqueous electrolyte solutions: Part 5. Potassium bromide and potassium iodide 0.1, 0.5, and 1.0 mol kg− 1 solutions at temperatures from T= 278.15 to 338.15 K. Journal of Molecular Liquids.

[28] Ali K, Bilal S (2009). Surface tensions and thermodynamic parameters of surface formation of aqueous salt solutions: III. Aqueous solution of KCl, KBr and KI. Colloids and Surfaces A: Physicochemical and Engineering Aspects.

[29] Jones G, Hartmann ML (1915). A study of the system: water, potassium iodide and iodine at zero degrees. Journal of the American Chemical Society.

[30] Withers PJ (2021). X-ray computed tomography. Nature Reviews Methods Primers.

[31] Dierick M (2014). Recent micro-CT scanner developments at UGCT. Nuclear Instruments and Methods in Physics Research Section B: Beam Interactions with Materials and Atoms.

[32] Nieuwenhuizen RPJ (2013). Measuring image resolution in optical nanoscopy. Nat. Methods..

[33] Schindelin J (2012). Fiji: An open-source platform for biological-image analysis. Nat. Methods..

[34] Angelidakis V, Nadimi S, Utili S (2021). SHape Analyser for Particle Engineering (SHAPE): Seamless Characterisation and Simplification of Particle Morphology from Imaging Data. Computer Physics Communications.

